# Comparative Investigation on the Soil Burial Degradation Behaviour of Polymer Films for Agriculture before and after Photo-Oxidation

**DOI:** 10.3390/polym12040753

**Published:** 2020-03-30

**Authors:** Francesco Paolo La Mantia, Laura Ascione, Maria Chiara Mistretta, Marco Rapisarda, Paola Rizzarelli

**Affiliations:** 1 Dipartimento di Ingegneria, Università di Palermo, Viale delle Scienze, 90128 Palermo, Italy; mc.mistretta@gmail.com; 2 Consorzio Interuniversitario Nazionale per la Scienza e Tecnologia dei Materiali (INSTM), Via Giusti 9, 55100 Firenze, Italy; ascione.laura@gmail.com; 3 Istituto per i Polimeri, Compositi e Biomateriali, Consiglio Nazionale delle Ricerche, Via Paolo Gaifami 18, 95126 Catania, Italy; marcorapis7@gmail.com

**Keywords:** biodegradable polymers, mulch films, soil burial test, Ecovio^®^, Mater-Bi^®^, polylactide, poly(butyleneadipate-*co*-butyleneterephtalate), polyethylene, polymer degradation, photo-oxidation

## Abstract

Polymer films based on biodegradable polymers, polyethylene (PE) and modified PE with oxo-degradable additive were prepared by film blowing. Carbon black (1%) was added to all the films. Commercial biodegradable Ecovio^®^ and Mater-Bi^®^ samples were used. Mechanical properties, soil burial degradation and surface wettability were investigated, before and after UV irradiation. Chemical modifications induced by UV and soil degradation, or a synergic effect, were highlighted by Attenuated Total Reflection-Fourier Transform Infra-Red (ATR-FTIR). Photo-oxidized film samples with an elongation at break equal to 50% and 0.5 the initial value were selected for the soil burial degradation test at 30 °C. Weight loss measurements were used to follow biodegradation in soil. Predictably, the degradation in soil was higher for biodegradable polymer-based films than for the PE-based ones. UV irradiation increased surface wettability and encouraged the disintegration in soil of all the samples. In fact, photo-oxidation produced a molar mass reduction and hydrophilic end groups, thus increasing surface erosion and weight loss. This paper not only supplies new criteria to evaluate the performance of biodegradable films in agriculture, before and after lifetime, but also provides a comparative analysis on the soil burial degradation behaviour with traditional ones.

## 1. Introduction

Polymer films are widely applied in agriculture [1]. The main applications of plastic films are for greenhouses and mulching. The more extensively used polymers for these applications are polyethylenes (LDPE and LLDPE) and the copolymer poly(ethylene-vinyl acetate) (EVA). The importance of these films consists in the protection of the cultures and creation of a microclimate that strongly improves the yield of the plants. The principal problem of these films is their short, or relatively short, life and the consequent frequent necessity to eliminate them in huge amounts from the ground. Indeed, the photo-oxidation strongly worsens the mechanical and optical properties of the polymers and, nevertheless the strong stabilizing systems used, the lifetime is no more than three-four years. Anyway, at the end of the service life, all these films must be collected to avoid the dissemination in the ground with a negative environmental impact [2]. Recycling of these films is relatively simple because of the easy differentiated collection for the similar composition. However, the properties of the recycled materials are relatively poor for the photo-oxidation undergone during their use. The recycling is more complicated for the mulch films as their very low thickness (15–50 micron) implies many difficulties during their washing and the fragmentation. For these films, then, the use of biodegradable, compostable polymers could be a good opportunity [3,4]. The films are mixed with the soil and buried in the ground during the ploughing. The successive biodegradation process gives rise to compost that is a fertilizer for the ground.

Aliphatic polyesters are the most adopted biodegradable materials in food packaging as well as in agriculture and medical fields [5]. Diverse studies on commercial biodegradable polymers have been performed to verify their performance in mulching or irrigation pipes [4,6,7]. Nevertheless, both plastic mulch films and irrigation pipes, during their service life, are exposed to sunlight. Thus, the occurrence of photo-aging can modify their properties and performances as well as their biodegradation rates.

The degradation of plastic materials, traditional and biodegradable, involves different processes promoted by one or more environmental factors (i.e., heat, light, microorganisms) or chemicals. The degradation produces irreversible structural changes that are mostly unfavourable or, in some cases, necessary, as in biodegradation processes, or are further generated to determine polymer structure, such as in pyrolysis-gas chromatography-mass spectrometry (Py-GC/MS) studies [8,9].

The degradability in soil of poly(vinyl alcohol) [10], copolyesters [11] and poly(ester amide) [12] film samples has been investigated under controlled soil burial conditions. However, the influence of UV on degradation rates in soil has been limited investigated. In fact, despite extensive research on biodegradable materials, only some studies have been aimed on polymer degradation related to a synergistic effect of UV exposure and soil burial [6,7,13,14,15,16]. Briassoulis et al. evaluated the degradation behaviour of MaterBi^®^-based films and irrigation tubes under real field conditions [6,7]. Kijchavengkul et al. studied the degradation of an aliphatic-aromatic biodegradable polyester film under solar exposure and soil burial in a tropical area [13]. Recently, we investigated the photo-oxidative and soil burial degradation of irrigation tubes based on biodegradable polymer blends under controlled conditions [14]. Actually, laboratory tests supply more reproducible data for both degradation mechanisms and kinetics.

It should be always kept in mind that biodegradation is schematically a three step process: in the first one, macromolecular chains are depolymerized into monomers and oligomers; in the second stage, the monomers and oligomers are taken up as biomass; and finally in the third step, the respiration of biomass there consumes O_2_ and produces CO_2_ and H_2_O (under aerobic conditions). The measurement of the plastic material consumption does not allow to ascertain whether the process has actually been completed or has prevented, for example, at depolymerisation. As a result, all the standardized methods for determining biodegradation are focused on the measurement of respiration, i.e., the conversion into CO_2_ of the carbon initially present in the plastic through the use of the oxidant (O_2_). On the other hand, most of the papers in the literature concerning polymer and composite biodegradation are based on weight loss [17,18,19] that is considered a measurement of plastic film biodegradability.

Aim of this work is to study the degradation behaviour in soil of a couple of biodegradable polymer systems after a photo-oxidative aging that simulates the real conditions of the service life of the film samples to be used in agriculture. For comparison, films of conventional polyethylene (PE) and PE-modified with an oxo-biodegradable compound have been used. A very interesting issue of the present work is that the films have not been aged under UV irradiation for the same time before the soil burial test. In particular, they have been aged until the elongation at break (EB) reached a given value, i.e., when the EB arrived at one half of the initial value, according to an international rule for the use of much films [20], as well as at the value of 50%. The elongation at break has been chosen to monitor the degradation of these films because it is a property of the polymers very sensitive to the changes of structure and morphology. In addition, it is very important for the use of these films as they must remain flexible during their use.

By blow film extrusion, we have prepared film samples based on biodegradable polymers (Ecovio^®^ and Mater-Bi^®^), PE and PE modified with oxo-degradable additive. Soil burial tests were carried out and weight loss used to monitor biodegradation rate. Mechanical properties determined. Remarkably, the films have been photo-oxidized up to the EB reached the value of 50% and one half of the initial value. We compared mechanical properties, weight loss due to burial degradation in soil and surface wettability, before and after UV irradiation. Structural modifications induced by UV and soil degradation, or a synergic effect, were checked by Attenuated Total Reflection (ATR)-FTIR.

## 2. Materials and Methods

### 2.1. Materials

The samples investigated in this work were three films produced in our laboratory and a commercial one. Sample codes of the films, thickness and extrusion temperature are reported in Table 1. PE is a film made by a blend of 80% LDPE (FC 30, Versalis, San Donato Milanese, Italy) and 20% LLDPE (FG20, Versalis); this composition has been chosen because is used in the manufacturing of mulch films. PE-OXO is made by the same polymer blend by adding 1% wt/wt of an oxo-biodegradable masterbatch, d2w (Symphony Environmental, Hertfordshire, UK). B1 is a film prepared by ECOVIO F23B1 (Bayer, Leverkusen, Germany), a biodegradable blend poly(butyleneadipate-*co*-butyleneterephtalate) (PBAT) with small amount of polylactide (PLA), containing about 12% of insoluble, inert particles. This amount has been determined after a Soxhlet extraction in tetrahydrofuran. B2 is a commercial film based on Mater-Bi EF04P by Novamont. This biodegradable polymer system is a blend constituted by corn starch and a biodegradable thermoplastic copolyester, Origo BI (Novamont, Novara, Italy), containing about 8% of insoluble, inert particles. Carbon black (1%) was added to all the films. The first three films were produced in a Brabender single screw extruder (D = 19 mm, L/D = 25) equipped with a head for film blowing.

### 2.2. Methods

#### 2.2.1. Accelerated Weathering Tests

The films were exposed to accelerated weathering tests in a Q-UV Panel system (Q-Labs Corp., Westlake, OH, USA) containing eight UVB lamps with a peak at 313 nm. The accelerated weathering tests were carried out using continuously the following cycle: a period of UV irradiation for 8 h at 70 °C followed by a steam condensation (deionized water spray) for 4 h at 55 °C. Film samples were removed from the chamber at different aging intervals.

#### 2.2.2. Mechanical Properties

Stress-strain curves were measured using a universal testing machine mod. 3365 (Instron, Norwood, MA, USA). The elastic modulus was measured at a speed of 1 mm/min until the deformation was 10%. Then, the crosshead speed was increased to 100 mm/min until the specimen breaks. The values of elastic modulus, E, tensile strength, TS, and elongation at break, EB, were calculated as average of 10 experimental values.

#### 2.2.3. Attenuated Total Reflection-Fourier Transform Infra-Red (ATR-FTIR)

ATR-FTIR spectra were measured by using a Spectrum Two spectrometer (Perkin-Elmer, Waltham, MA, USA) with the Spectrum software. Spectra were recorded with 8 scans and 4 cm^−1^ resolution. In order to compare the different effects on the structures of photo-oxidation and degradation in soil, the carbonyl index (CI) was evaluated for the virgin and degraded samples. In particular, the CI was calculated from the ratio of the total area of the absorption bands between 1808–1550 cm^−1^ or 1801–1487 cm^−1^ (carbonyl region) and 3029–2749 cm^−1^ (stretching of the CH_2_) respectively for the B1 and B2 samples, from the ratio of the total of the peaks in the range 1826–1550 cm^−1^ (carbonyl region) and that 2983–2662cm^−1^ (stretching of the CH_2_ and CH_3_ groups) for the PE based films.

#### 2.2.4. Soil Burial Test

Tests were carried out at 30 ± 0.1 °C, under moisture-controlled conditions. Triplicate specimens of film samples were placed in darkened vessels containing a multi-layer substrate [12]. Filter paper and polyethylene samples were used, respectively, as a positive and negative control. Film portions of 2 cm × 2 cm were cut. Specimens of film (initial weight 7–8 mg, filter paper ~29 mg; Mettler Toledo MX5, *d* = 1 µg) were sandwiched between two layers of a mixture of milled perlite (50 g) and commercial soil (200 g), moistened with 100 mL of distilled water. The bottom and top layers were filled with 60 g of perlite moistened with 120 mL of distilled water. Perlite was used for increasing aeration to the soil and the amount of water retained. A flow of moistened air was supplied from the bottom of each vessel every 24 h for 15 min. Film samples were removed after regular intervals, brushed softly, washed with distilled water several times and dried under vacuum in the presence of P_2_O_5_ at room temperature, to constant weight [21]. The degree of degradation was evaluated by weight loss (WL) by using the following equation:

WL (%) = (Wi − Wt)/Wi × 100,(1)

where Wi is the initial weight of the sample and Wt is the weight after the established time.

#### 2.2.5. Contact Angle

The surface wettability values of samples were measured at room temperature using a contact angle goniometer (OCA15EC, Dataphysics, Filderstadt, Germany). Static contact angle (CA) were determined dropping 2 μL of water from a micro syringe onto the film surfaces and analysing by software (SCA 20) the images taken by the connect video camera. To eliminate interference, the sample was previously equilibrated for 30 min and then CA was measured. The films were kept flat using a film sample holder that allowed their correct positioning and stretching. Five measurements were carried out for each sample in order to ensure repeatability of the experiments.

## 3. Results

### 3.1. Mechanical Properties

**Figure 1:**
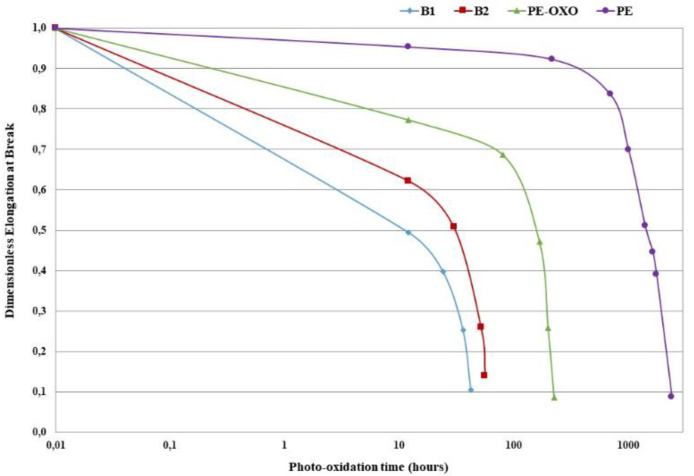
Dimensionless elongation at break vs. photo-oxidation time of all the samples.

The values of the elongation at break (EB_0_), tensile strength and elastic modulus of the virgin films are listed in Table 2. The dimensionless elongation at break vs. photo-oxidation time curves of all the samples are reported in Figure 1. The dimensionless values of the elongation at break were obtained by dividing the values of the elongation at break recorded for each photo-oxidation time by the corresponding initial value (EB_0_).

Figure 1 highlights that the photo-oxidation affects the elongation at break of the two biodegradable polymers much more than that of the PE-based film samples. Of course, the presence of a pro-oxidant system strongly increases the kinetic of photo-oxidation of the PE matrix. As for the two biodegradable polymers, the photo-oxidation kinetics are slightly more rapid for the B1 samples.

**Figure 2:**
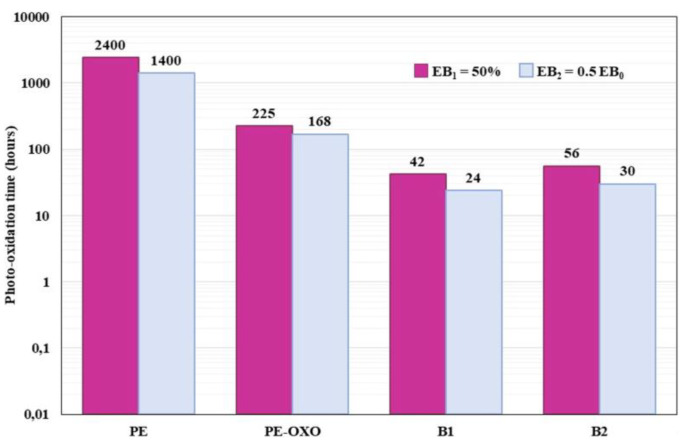
Photo-oxidation times (hours) of different samples when the elongation at break reaches the value of 50% (EB_1_) and one half of the initial value (EB_2_).

In Figure 2 the photo-oxidation times at which all the films reach the elongation at break equal to 50% (EB_1_) and to one half (EB_2_) of the initial value (EB_0_) are reported.

From this plot, it is well evident as the useful accelerated lifetime of the biodegradable films is significantly shorter than that of the PE samples. In particular, considering the values of the elongation at break 50% as the lowest value compatible with the use, the lifetime—as measured in these accelerated aging experimental conditions—of the PE sample is about 2400 h, while it is 42 and 56 h for the two biodegradable polymer systems. The presence of the pro-oxidant additive in the PE matrix considerably reduces the useful lifetime to about 225 h.

### 3.2. Soil Burial Degradation

Soil burial degradation tests were carried sandwiching the polymer films between two layers of a mixture of milled perlite and commercial soil to simulate soil degradation after their use lifetime. Perlite was used to increase the amount of water retained and accelerate degradation in soil.

Figure S1 summarizes the photo-oxidation and soil burial degradation intervals, together with some representative photographs of the film samples recovered after the burial test. Except for the B2 sample photo-oxidized (56 h), all the film portions appeared almost intact.

**Figure 3:**
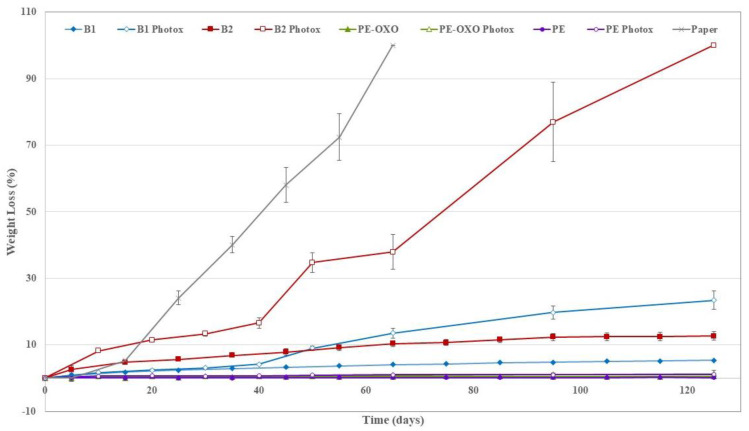
Average weight loss values (%) vs. degradation time for all the film samples—virgin and photo-oxidized.

In Figure 3 the average weight losses of the virgin and photo-oxidized samples are reported vs. the soil burial time. Of course, the WL increases and kinetic of biodegradation decreases with burial degradation time. Additionally, the WL values increased because of UV exposure, as recently evidenced in soil burial degradation of photo-oxidized irrigation tubes based on biodegradable polymer blends [14]. Reasonably, UV exposure yielded a molecular weight (MW) reduction producing oligomeric chains, bearing hydrophilic chain ends, which are more easily removed from the film surface and susceptible to the attacks of microorganisms in the soil. In fact, it must be kept in mind that biodegradation proceeds in three stages. Whenever WL is used to monitor the degradation of polymer samples, just the first step is involved, i.e., macromolecular chain depolymerisation into monomers and oligomers that are eroded from the surface. UV irradiation enhances and accelerates the formation of monomers and oligomers, via random main chain scission, increasing consequently the WL rate.

**Figure 4:**
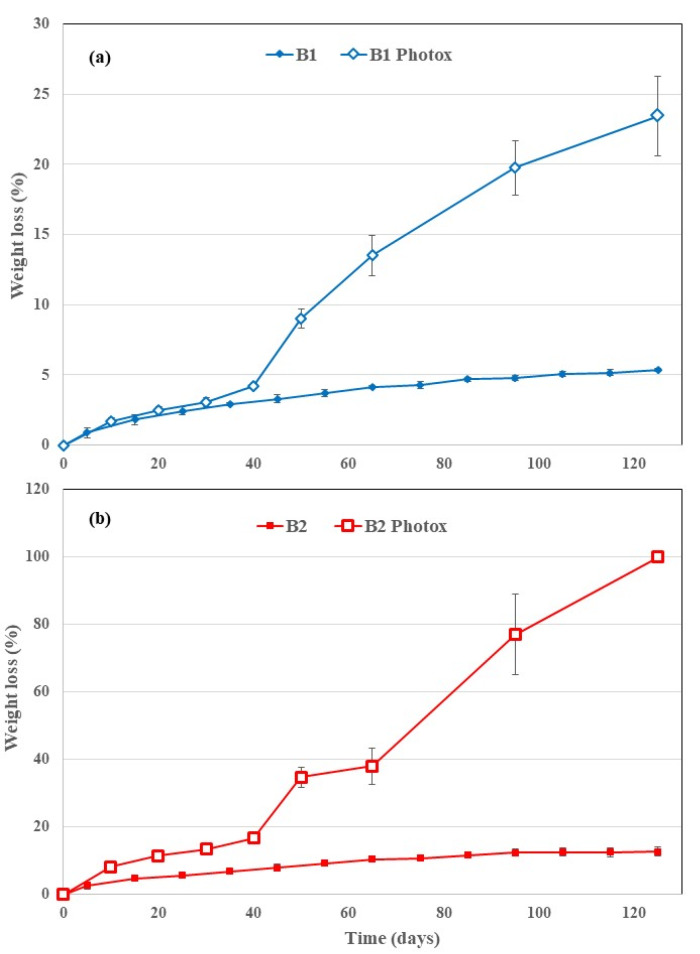
Average weight loss values (%) vs. degradation time for the film samples (**a**) B1 and (**b**) B2, virgin and photo-oxidized.

**Figure 5:**
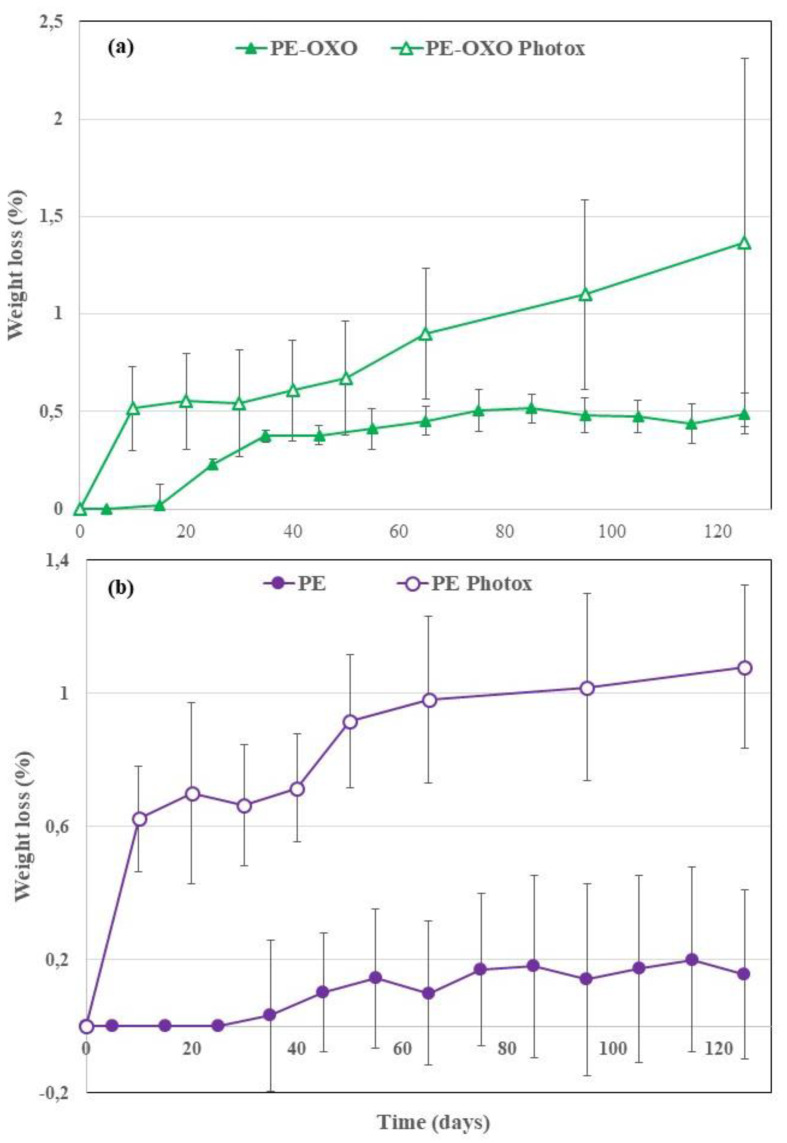
Average weight loss values (%) vs. degradation time for the film samples (**a**) polyethylene (PE)-OXO and (**b**) PE, virgin and photo-oxidized.

The positive control (paper) and B2 sample photo-oxidized for 56 h were totally disintegrated (WL = 100%) respectively after 65 and 125 days of soil burial test (Figure 3). The complete disintegration in soil of the B2 sample photo-oxidized for 56 h suggests that the photo-oxidation is not limited to the surface but occurred throughout the matrix bulk, in agreement with the literature [13]. WL values of B1 (Figure 4a) are remarkably lower than that of B2 (Figure 4b) samples. The photo-oxidized samples of all the films show a more pronounced WL than the corresponding virgin samples (Figure 4 and Figure 5). This holds also for the PE-based films, but the final values of the WL are almost negligible although the photo-oxidized PE-OXO film (Figure 5a) shows WL values slightly larger than that of the pure PE (Figure 5b). On the contrary, the weight losses of the two biodegradable samples are quite similar for the virgin films. WL curves of B1 and B2 photo-oxidized samples shows a comparable trend with an induction period (40 days) and then a rapid increase.

### 3.3. Wettability of the Film Surfaces

**Figure 6:**
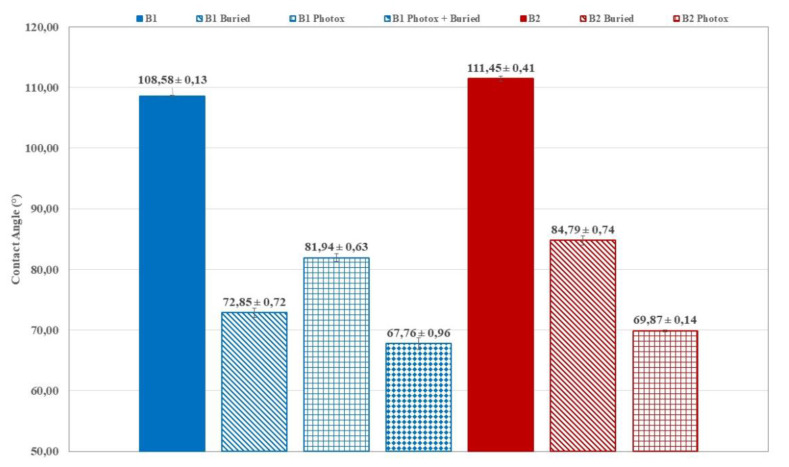
Average contact angle values for the film samples B1 and B2, virgin, buried (125 days) and photo-oxidized. After 125 days of soil burial test, photo-oxidized B2 samples were completely disintegrated and it was not possible to carry out the contact angle (CA) measurements.

**Figure 7:**
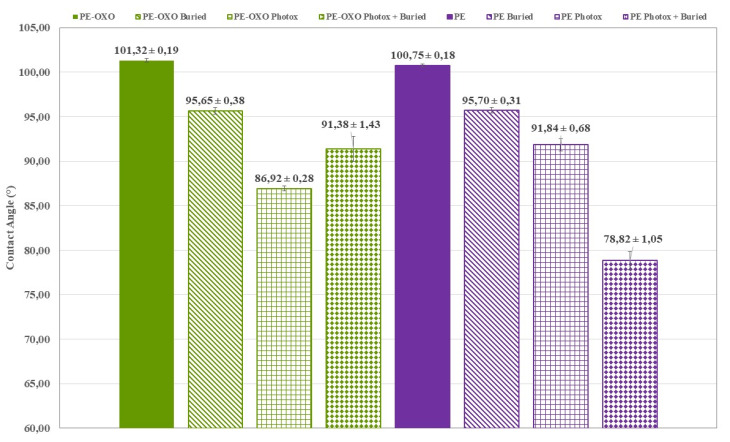
Average contact angle values for the film samples PE-OXO and PE, virgin, buried (125 days) and photo-oxidized.

The wettability of the film surfaces submitted to UV irradiation was determined by contact angle measurements. In fact, chemical modifications on the surface of the thin films, induced by UV irradiation and/or soil degradation, increase plastic samples wettability, highlighted by CA decrease (Figure 6 and Figure 7) and, consequently, the microbial susceptibility is encouraged. The UV induced increase in surface wettability is more pronounced for photo-oxidized and buried B1 (Figure 6) samples (CA ~68°) as well as for photo-oxidized B2 films (CA ~70°, Figure 6). CA decrease is reasonably related to the formation of hydrolytic chain ends. In photo-oxidised and buried B1 samples the CA is quite similar to that of B1 buried (Figure 6). This could be related to the surface erosion and the removal of the oligomeric species produced by hydrolysis.

In a similar way, the increase in the wettability of the irradiated PE (Figure 7) and PE-OXO (Figure 7) samples can be attributed to the formation of hydrophilic groups on the polymer surface by photo-oxidation, which occurs by the exposure of PE to UV irradiation in the presence of air [22,23]. The photo-degradation of PE samples, promoted by the oxo-degradable additive, induces a more marked decrease in the contact angle because of the introduction of carbonyl groups (Figure 7). However, wettability is higher in photo-oxidized and buried PE sample (CA ~79°, Figure 7) than in photo-oxidized and buried PE-OXO (CA ~91°, Figure 7). This can be reasonably due to the higher WL and thus faster surface erosion of oligomers, bearing hydrophilic chain ends, in the PE-OXO (Figure 5a) than in PE sample (Figure 5b).

### 3.4. UV and Soil Burial Induced Modifications

Both UV and soil burial degradation induced chemical modifications on the surface of all the polymeric films. Then, to check the structural changes caused by the different types of degradation, ATR-FTIR analysis was performed on virgin, photo-oxidized, soil buried and photo-oxidized + buried film samples. In Figures S2 and S3, ATR-FTIR spectra are reported as examples of the trend of variations in functional groups on the film surface. Overall, degradation produced a broadening and a change of the peak area more noteworthy in B2 (Figure S2b) and PE (Figure S3b) samples compared respectively with B1 (Figure S2a) and PE-OXO (Figure S3a). A significant decrease in peak intensity between photo-oxidized and photo-oxidized + buried B2 sample was more evident (Figure S2b). Remarkably, it is in accordance with the higher surface erosion of oligomers produced by UV irradiation and the resultant weight loss values increase.

**Figure 8:**
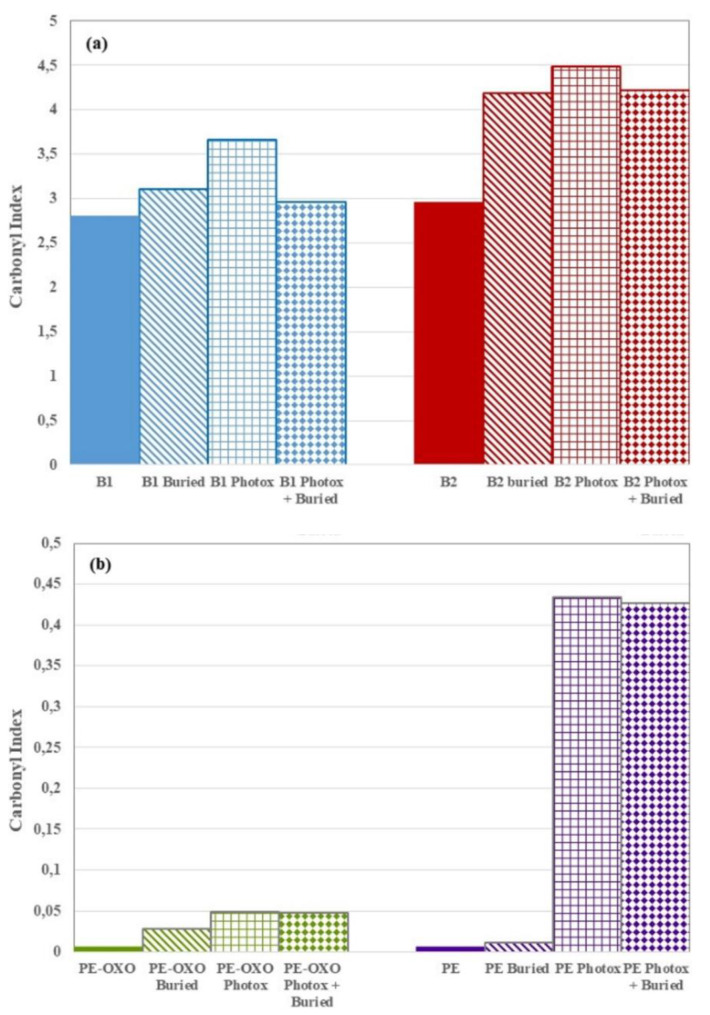
Carbonyl index values for (**a**) the two biodegradable and (**b**) PE based film samples. CI (B1) = A(1808–1550 cm^−1^)/A(3029–2749 cm^−1^); CI (B2) = A(1801–1487 cm^−1^)/A(3029–2749 cm^−1^); CI (PE and PE-OXO) = A(1826–1550 cm^−1^)/A(2983– 2662 cm^−1^). B1 = Ecovio^®^-based and B2 = Mater Bi^®^-based films.

The increase of the carbonyl groups was a marker of the photo-oxidation evolvement as well as of hydrolytic reactions taking place during soil degradation. Unsurprisingly, in agreement with the literature [13,14], the spectra of the two biodegradable polymer-based films show that both photo-oxidation and degradation in soil increase the CI (Figure 8a). Remarkably, the area ratio of photo-oxidized + buried B1 and B2 sample is lower than that photo-oxidized and similar to the not photo-oxidized and buried ones. This result suggests that the oligomers, bearing carbonyl groups, have been partially removed from the surface during the soil burial test. However, B1 films were clearly more vulnerable to photo-oxidation than to hydrolysis; photo-oxidation products were removed by degradation in soil and CI of “B1 photox + buried” was lower than “B1 photox” sample (Figure 8a). Compared to the virgin samples, the increase of CO functional groups, due to the combined photo-oxidation and soil degradation, was significantly more evident in the B2 than in the B1 samples; in fact, the UV irradiation did not accelerate the WL rate as it did in the B2 films (Figure 4). These results were in agreement with the degradation process and the choice of monitoring degradation in soil through weight loss. Actually, the B2 films were much more prone than the B1 ones, since monomers and low-molecular-weight oligomers were originated and removed in the erosion step faster than in the B1 sample.

The carbonyl index values show similar trends in PE-OXO and PE samples. Photo-oxidation provides an increase in the area ratio, significantly more marked for PE than for PE-OXO (Figure 8b), due to the formation of ketones, aldehydes and/or esters groups. The successive degradation in soil on photo-oxidized PE films produces a low CI decrease reasonably related to a limited surface erosion of oligomers highlighted by WL (Figure 5b). The small CI decrease in photo-oxidized PE-OXO and PE after soil burial test for 125 days is in agreement with the literature and was attributed to a minimal consumption of CO groups by microorganisms [23].

It has to be underlined that carbon back is a UV absorber and its photo-stabilizing action is due to the absorption of the ultraviolet energy that, then, is no more available for the formation of radicals and for the consequent propagation of the oxidation reactions [24]. This means that carbon black does not interfere with the photo-oxidative mechanisms of the different matrices and the effect is just to shift towards higher times the photo-oxidation increasing the induction time of the process. Carbon black slows down the molecular weight reduction and consequently the degradation process in soil. The shift towards higher time to starting photo-oxidation is relevant on a commercial point of view above all with biodegradable polymeric systems whose performance can be impaired by photo-oxidation processes more than the traditional ones [4].

## 4. Conclusions

A comparative investigation on the degradation behaviour in soil of traditional and biodegradable polymer films for agriculture, before and after photo-oxidation, have been accomplished. The two polyethylene-based samples showed the better mechanical properties, while the Ecovio^®^-based films, named B1, displayed a lower value of the tensile strength and the Mater-Bi^®^-based films, called B2, had the lowest values of the elastic modulus and elongation at break. However, all the samples showed high values of the elongation at break that strongly decreases with the photo-oxidation time, in particular for the biodegradable films. Interestingly, the film samples have been aged under UV irradiation until the elongation at break reached the value of 50%, according to an international rule for the use of much films, as well as at one half of the initial value.

Soil burial degradation tests were carried sandwiching the samples, virgin and photo-oxidized, between two layers of a mixture of milled perlite and commercial soil to simulate degradation in soil after their use lifetime as mulch films. Weight loss measurements showed that the Mater-Bi^®^-based films were more susceptible to soil degradation than the Ecovio^®^-based ones were. Additionally, for all of the samples, soil degradation was accelerated by UV irradiation. The positive control (paper) was biodegraded in soil within 65 days, while photo-oxidized Mater-Bi^®^-based films were fully disintegrated in soil after 125 days at 30 °C. These results suggest that the photo-oxidation was not restricted to the surface but occurred throughout the bulk. Chemical structure changes induced by UV exposure affected wettability of films, as highlighted by contact angle decrease. A relevant increase intensity in carbonyl peaks and carbonyl index was highlighted in photo-oxidized biodegradable and PE film samples. Carbonyl index values of photo-oxidized biodegradable and PE film samples decreased after soil burial test because of surface erosion of monomers and oligomers.

This paper not only supplies new criteria to evaluate the performance of biodegradable films in agriculture, before and after lifetime, but also yields a comparative analysis on the soil burial degradation behaviour with traditional ones. However, accelerated weathering test does not provide real lifetime. Further studies will be addressed to establish a correspondence between accelerated weathering time and aging time in real conditions.

## Figures and Tables

**Table 1:** Sample code, extrusion temperature of the three samples produced in laboratory scale and thickness for all the investigated films.

Sample Code	Film Thickness (μm)	Extrusion Temperature (°C)
PE	20	180
PE-OXO	20	180
B1	13	190
B2	15	---

**Table 2:** Mechanical properties of the virgin films.

Sample Code	Elastic Modulus, E (MPa)	Tensile Strength, TS (MPa)	Elongation at Break, EB_0_ (%)
**PE**	206 ± 19	19.5 ± 1.1	586 ± 12
**PE-OXO**	183 ± 23	15.9 ± 1.4	582 ± 32
**B1**	186 ± 16	8.6 ± 2.2	460 ± 11
**B2**	129 ± 6	21.1 ± 2.0	392 ± 22

## Supplementary Materials

The following are available online at https://www.mdpi.com/2073-4360/12/4/753/s1.
