# Supplementary material for: Comparative Investigation on the Soil Burial Degradation Behaviour of Polymer Films for Agriculture before and after Photo-Oxidation
Source: Polymers (Basel). 2020 Mar 30;12(4):753. doi: 10.3390/polym12040753 (PMC7240387; doi:10.3390/polym12040753)
Supplement: Supplementary file 1 [file polymers-12-00753-s001.pdf]

Supplementary Materials

Comparative Investigation on the Soil Burial Degradation Behaviour of Polymer Films for Agriculture before and after Photo-Oxidation

Francesco Paolo La Mantia [1,2,*], Laura Ascione [2], Maria Chiara Mistretta [1], Marco Rapisarda [3] and Paola Rizzarelli [3,*]

Sample	Photo-oxidation time (h)	Soil burial degradation time (days)	
B1	0	125	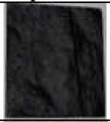
	40	125	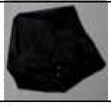
B2	0	125	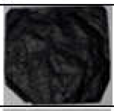
	56	125/95	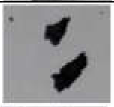 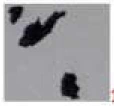 [1]
PE-OXO	0	125	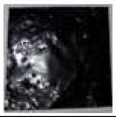
	168	125	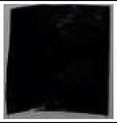
PE	0	125	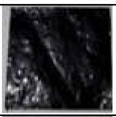
	2400	125	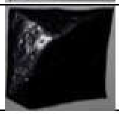

Figure S1. Photo-oxidation and soil burial degradation intervals, and some representative photographs of the film samples recovered after the burial test. [1]After 125 days of soil burial test, B2 samples were completely disintegrated. Photographs refer to fragment recovered after 95 days of degradation in soil.

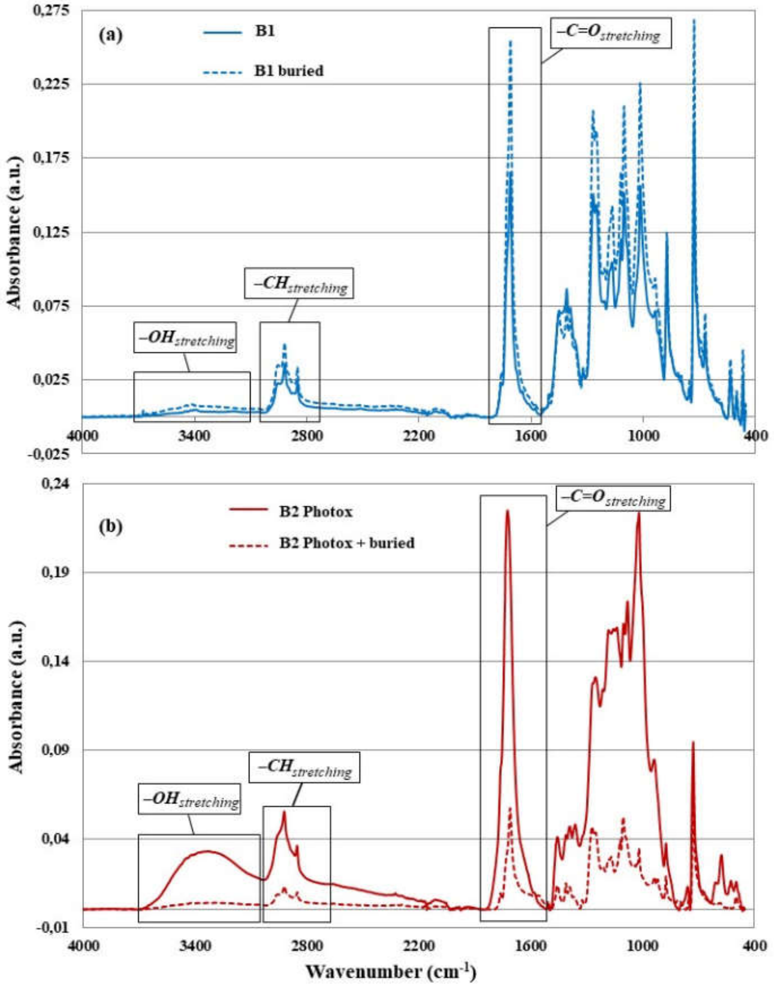

Figure S2. Examples of selected Attenuated Total Reflection (ATR)-FTIR spectra: **(a)** virgin and buried B1 sample; **(b)** photo-oxidized and photo-oxidized + buried B2 sample. B1 = Ecovio®-based and B2 = commercial Mater Bi®-based films. After 125 days of soil burial test, B2 samples, photo-oxidized 56 h, were completely disintegrated. ATR-FTIR spectrum refers to B2 samples, photo-oxidized 30 h and recovered after 125 days of degradation in soil.

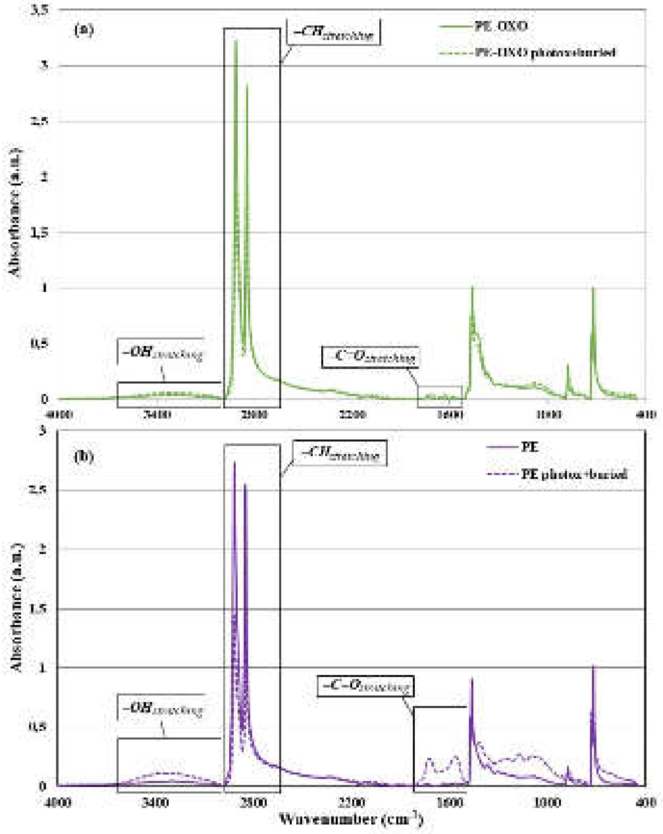

Figure S3. Examples of selected ATR-FTIR spectra: photo-oxidized and photo-oxidized + buried **(a)** PE-OXO and **(b)** PE samples.

© 2020 by the authors. Submitted for possible open access publication under the terms and conditions of the Creative Commons Attribution (CC BY) license (http://creativecommons.org/licenses/by/4.0/).
